# A Normalized Shear Deformation Indicator for Ultrasound Strain Elastography in Breast Tissues: An* In Vivo* Feasibility Study

**DOI:** 10.1155/2018/2053612

**Published:** 2018-02-12

**Authors:** Jingfeng Jiang, Bo Peng

**Affiliations:** ^1^Department of Biomedical Engineering, Michigan Technological University, 1400 Townsend Drive, Houghton, MI 49931, USA; ^2^Department of Medical Physics, University of Wisconsin-Madison, Madison, WI 53705, USA; ^3^School of Computer Science, Southwest Petroleum University, Chengdu 610500, China

## Abstract

The shear deformation under loads contains useful information for distinguishing benign breast lesions from malignant ones. In this study, we proposed a normalized shear deformation indicator (NSDI) that was derived from the concept of principal strains. Since the NSDI requires both high-quality axial and lateral (parallel and perpendicular to the beam, resp.) displacement estimates, a strategy combining high-quality speckle tracking with signal “denoising” was employed. Both techniques were previously published by our group. Finite element (FE) models were used to identify possible causes for elevated NSDI values in and around breast lesions, followed by an analysis of ultrasound data acquired from 26 biopsy-confirmed* in vivo* breast lesions. We found that, theoretically, the elevated NSDI values could be attributed to two factors: significantly hardened tissue stiffness and increasing heterogeneity. The analysis of* in vivo* data showed that the proposed NSDI values were higher (*p* < 0.05) among malignant cancers as compared to those measured from benign ones. In conclusion, our preliminary results demonstrated that the calculation of NSDI value is feasible and NSDI could add value to breast lesion differentiation with current clinical equipment as a postprocessing tool.

## 1. Introduction

According to the US National Institute of Cancer, an estimated 252,710 new cases of invasive breast cancer are expected to be diagnosed in 2017. In light of the widespread use of the ultrasound, American College of Radiology has developed a BI-RADS lexicon to standardize the characterization of breast lesions under ultrasound [1]. Through analyzing BIRADS 3–5 lesions, Hille et al. reported that the sensitivity and specificity were 92% and 85%, respectively [2]. Their result suggested that ultrasound probably should not be used alone as the first line of imaging.

In the last two decades, a lot of efforts have been devoted to ultrasound strain elastography (SE) [3]. In Ultrasound SE, tissue displacements are first tracked by correlating radio frequency (RF) signals before and after compression. Then, axial (parallel to the acoustic beam direction) strain defined by the change in length divided by the length before compression can be used as a surrogate for relative tissue elasticity. Ultrasound SE has been successfully applied to noninvasive differentiation of breast tumors [4–7] with several identified metrics: area ratio, elasticity score, strain ratio, and length ratio. The first metric is known as the area ratio which was defined as the ratio between the tumor area measured from the axial strain elastogram and the tumor area appearing on the B-mode image [4, 5, 7]. Typically, a large area ratio (e.g., >1.0) is correlated to an increasing possibility of malignancy. The second metric used a scoring system [6], in which the overall tumor appearance on the axial strain elastogram was rated between 1 and 5 based on a set of graphic criteria. The strain ratio between the tumor and a selected region containing background tissue was also adopted by numerous studies [4, 8, 9]. The fourth metric is the length ratio. The length ratio is defined as the lesion length measured from the axial strain elastogram over that which appeared on the B-mode image. Based on several published meta-analyses [10–12], the sensitivity of these four metrics often varied from 80% to 98%, while the specificity typically ranged from 85% to 95%. Considerable inter- and intraobserver variability was also reported [13].

Continued research efforts have been devoted to improving the efficacy of ultrasound SE. Excellent work was done by Dr. Thittai and colleagues [14, 15] to use shear information (i.e., the shape change) for the breast lesion differentiation. Recall that previously discussed four metrics were derived from the axial strains only reflecting the dimensional changes under the external compression. The shear strain is defined as follows [16]:

(1) $$\begin{array}{c} \tau_{xy}=\frac{1}{2}\left(\;\frac{\partial u}{\partial x}+\frac{\partial v}{\partial y}\;\right), \end{array}$$

where *u*, *v*, *x*, and *y* are the axial and lateral displacements and lateral and axial spatial coordinates, respectively. In the literature, studies [9, 15] stipulated that shear strains could be useful in terms of characterization of the lesion mobility. Because of the poor quality among lateral displacements *v*, only the first component on the right-hand side of (1) was used. Thittai and colleagues named this technology axial-shear strain elastography. Although feasibility studies [9, 15] have demonstrated its usefulness, the axial-shear strain alone, theoretically, cannot be used as an indicator of shear deformation because it contains rigid-body rotation.

Normalization of axial-shear strain data has been attempted by others [17]. However, their approach was an ad hoc approach and only attempted to scale the axial-shear strain with the fitted local axial strain. Toward this end, the primary objective of this study was to develop an alternative but more rigorous method to assess the shear deformation based on the continuum mechanics. More specifically, the proposed normalized shear deformation indicator (NSDI) leverages the well-established concept of principle strain [16], requiring all three components of the 2D strain tensor: axial strain, lateral strain, and (full) shear strain. Consequently, the proposed NSDI metric requires both high-quality axial and lateral displacement estimates.

In order to improve lateral displacement quality, a published image denoising approach that enforces tissue incompressibility [18] was adopted for our convenience. Our denoising approach is conceptually similar to the work of Lubinski et al. [19] because both methods attempt to enforce the tissue incompressibility. However, main difference does exist. In the work of Lubinski et al., a laterally fixed central line within the tissue being imaged was required and such a laterally fixed line would be difficult to find from data acquired from* in vivo* tissues. In contrast, our denoising approach has no special requirement other than a two-dimensional ultrasonically estimated displacement vector field.

Toward this end, the primary objectives of this study are to (1) understand factors that influence the calculation of the NSDI metric through simplified finite element (FE) models and (2) demonstrate the feasibility of quantifying NSDI* in vivo*. The second objective was evaluated using* in vivo* breast ultrasound data acquired from biopsy-confirmed breast lesions [5].

## 2. Materials and Methods

### 2.1. Definition of Normalized Shear Deformation Indicator (NSDI)

Given the lateral strain *ϵ*_*xx*_, axial strain *ϵ*_*yy*_, and shear strain *ϵ*_*xy*_, *θ*_*p*_ below is an angle between the first principle strain *ϵ*_1_ and the positive direction of the lateral direction and can be evaluated as follows [16]:

(2) $$\begin{array}{c} \theta_{p}=\frac{1}{2}atan\left(\;\frac{2\epsilon_{xy}}{\epsilon_{xx}-\epsilon_{yy}}\;\right). \end{array}$$

When there is no presence of shear strain (i.e., *ϵ*_*xy*_ = 0), *θ*_*p*_ is equal to zero. With the increase of the shear strain *ϵ*_*xy*_, the absolute value of *θ*_*p*_ increases, indicating that the shear strain *ϵ*_*xy*_ plays a more prominent role. Eventually, under certain conditions (e.g., the pure shear condition *ϵ*_*xx*_ = *ϵ*_*yy*_ = 0), *θ*_*p*_ becomes *π*/4. Since the absolute value of *θ*_*p*_ ranges from 0 to *π*/4, *θ*_*p*_ can be normalized (hereafter referred to as normalized shear deformation indicator (NSDI)) as follows:

(3) $$\begin{array}{c} NSDI=\frac{\left|\theta_{p}\right|}{\pi /4}. \end{array}$$

Consequently, the NSDI metric represents a relative measure of the local shear deformation.

### 2.2. Implementation

There are three major steps in the proposed NSDI assessment; as stated before, methods from two of our previous publications [18, 20] were adopted for our convenience. In the first step, tracking* in vivo* tissue deformation was achieved through accumulations of smaller deformation as a multistep process [20, 21]. More formally, given a sequence of *N* ultrasound echo fields under a monotonic compression, sequential motion tracking was first performed between two adjacent frames using a published speckle tracking algorithm [20]. The tracking kernel size is approximately 1.5 mm (lateral; approximately one beam width) × 1.8 mm (axial; approximately 6 wavelength long at 7.5 MHz). Once all (*N* − 1) frame-to-frame displacement fields were obtained, all displacements were mapped to the coordinate system of the first ultrasound echo frame using B-spline interpolations [20] and then all spatially registered frame-to-frame displacements were summed to obtain the accumulated displacement estimates ($\tilde{u},\tilde{v}$) from the first frame to the *N*th frame. More details of this speckle tracking method can be found elsewhere [20]. Leveraging the availability of graphic processing units (GPUs), this algorithm has been implemented using a parallel computing platform CUDA (NVIDIA Inc., CA, USA).

In the second step, given a 2D displacement vector field $\left(\tilde{u},\tilde{v}\right)$ from a rectilinear domain *Ω*, obtaining a “regularized” displacement vector field (*u*, *v*) on *Ω* is equivalent to minimize the following energy function [18]:

(4) Fu,v=∫Ω∂u∂x+∂v∂y2dΩ+λ1∫Ωu~−u2dΩ+λ2∫Ωv~−v2dΩ,

where *λ*_1_ and *λ*_2_ are two positive parameters and are also known as the regularization constants. On the right-hand side of (4), the first item is the calculated incompressibility from the regularized displacement field (*u*, *v*), while the second and third items are two individual fidelity terms of the ultrasonically measured axial ($\tilde{u}$) and lateral ($\tilde{v}$) displacements, respectively. *λ*_1_ and *λ*_2_ control the trade-offs between the fidelity and the degree of tissue incompressibility. Details regarding solving (4) by the Euler-Lagrange variation of *F*(*u*, *v*) can be found in [18].

In the third step, the regularized displacement vector field (*u*, *v*) was used to estimate local strains, that is, *ϵ*_*xx*_ (lateral strain), *ϵ*_*yy*_ (axial strain), and *ϵ*_*xy*_ (shear strain). All three local strains were estimated using a low-pass-filter-based method [23] and windows used for axial and lateral strain estimation were both 1.8 mm. Finally, the proposed NSDI values were calculated and were used to form an image.

### 2.3. Finite Element Analysis

The 2D finite element analysis (FEA) was done using a commercial FEA package (ADPL version 17.0, ANSYS, Inc., Canonsburg, PA). Five different cases simulated along with their rationales are described below.

**Case 1 (varying deformation level).**

Typically, the tissue deformation under the freehand scanning from frame to frame varies [5]. In this study, varied levels of deformation occurring* in vivo* (0.25%–5%) were investigated.

**Case 2 (heterogeneity within the inclusion).**

A recent study [24] found that mechanical properties in and around breast cancers are more heterogeneous as compared to benign ones. This is consistent with cancer biology because cancer's microenvironment and the spatial distribution of the desmoplastic reaction are usually complex. Hence, the influence of these heterogeneities was investigated.

**Case 3 (varying the modulus ratio between the inclusion and the background).**

It is well known that pathological evolution of breast lesions influences their mechanical properties [25]. Measurements from 10* in vivo* breast lesions indicated that the (initial) shear modulus ratios between the lesion and the background approximately varied between 4 and 30 [26]. Thus, the modulus ratio was varied accordingly in a comparable range to investigate how this modulus ratio may influence the calculation of NSDI.

**Case 4 (varying inclusion size).**

Based on breast ultrasound, the size of breast lesions varies [27]. Thus, we decided to vary the diameter of the inclusion from 4 to 12 mm to understand how the size of the inclusion would affect the calculation of NSDI.

**Case 5 (varying connectivity between the inclusion and the background).**

Typically, clinical studies using axial-shear strain elastography found that axial-shear patterns among malignant cancers were different as compared to benign breast lesions [9, 15]. Prior studies have attributed the difference to the fact that benign breast tumors are often more loosely connected to the background and were felt by physicians as “bouncy.” Similar to the study conducted by Thitaikumar et al. [14], the friction coefficient was varied to quantify how the varying connectivity would affect the NSDI.

**Figure 1:**
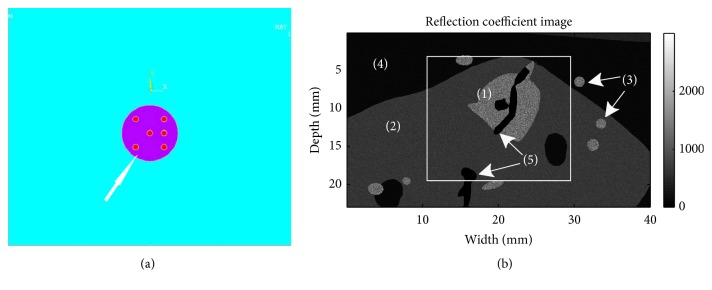
Illustrations of two FEA models: (a) a 2D heterogeneous inclusion model and (b) the middle “image” plane of a complex numerical breast phantom (i.e., lesion 2 phantom in a previous publication [22]). In (a), the arrow points to smaller harder inclusions inside the large inclusion. In (b), (1)–(5) denote lesion, fibroglandular tissue, Cooper's ligaments, breast fat, and necrotic zone, respectively.

In Cases 1 and 3–5, we simulated a circular hard inclusion embedded into a homogeneous background (40 mm by 40 mm) and this geometry was similar to the model used in [14]. In all 5 cases, displacement boundary conditions were applied. More specifically, all FEA models were compressed from the top for a fixed percentage and free to move on the sides (i.e., no lateral confinement). In the bottom boundary, the geometry was free to move along the lateral direction as well. Poisson's ratio value was set to 0.495 for both the background and inclusion. Contact elements were used to model the interface between the background and the inclusion. In the ANSYS software, friction coefficients of the inclusion and the background interface can be adjusted so that different degrees of bonding between the inclusion and the background can be achieved. In this study, friction coefficients of 0.1 and 1000 were used to represent a slipping boundary and a tightly connected/bonded condition, respectively. The friction coefficient of infinite corresponds to a fully bonded inclusion. In Case 2, five randomly positioned secondary inclusions (1.5 mm diameter and twice harder than the large 10 mm inclusion) were included as shown in Figure 1(a). More detailed descriptions of Cases 1–5 are summarized in Table 1.

3D FEA analysis was also performed using a complex numerical breast phantom (i.e., lesion 2 phantom in a previous publication [22]). Boundary conditions and material properties of the lesion 2 phantom were identical to those presented in the previous publication [22]. In order to keep the current study concise, interested readers are referred to that prior publication for details. Based on FEA-simulated displacements, NSDI values were also calculated for an “image” plane of the lesion 2 phantom (see Figure 1(b)).

### 2.4. Experimental Design

*In vivo* data with pathologically confirmed breast lesions were used to demonstrate the feasibility of utility of the NSDI metric in a clinical workflow. From an archived database of ultrasound scans of human breast lesions, 26 RF echo data sets were arbitrarily chosen. Among them, there were 13 cases of fibroadenoma (FA) and 13 cases of cancers (9 cases of invasive ductal carcinomas [IDC] and 4 cases of unspecified cancers). Once the motion tracking in a sequence was done, the accumulative strains approximately ranged from 0.5% to 15% (mean ± one standard deviation; 3.2% ± 3.0%) in those 26 cases. The detailed protocol for data acquisition was previously reported [5].

All data acquisition was approved by appropriate oversighting institutional review boards (IRBs) and patient consents were obtained. The IRB at the Michigan Technological University approved a secondary analysis of existing data. All* in vivo* data analyses including the manual lesion segmentation were done by a biomedical engineer who has approximately 15-year experience in strain elastography including algorithm development, data acquisition, and image analysis.

During the manual segmentation of a breast lesion, the operator first read a sequence of B-mode and strain images to decide the approximate location and contour of the breast lesion. The approximate location and contour of the lesion were used to set expectations of the lesion size and location. Then, B-mode and (axial and shear) strain images selected from that breast lesion were displayed side-by-side in MATLAB (MathWorks, Inc., MA, USA). Using image contrast provided by B-mode and strain images, the operator manually delineated the respective contours of the breast lesion. If there was little or no image contrast around a part of the lesion boundary, the operator would use a smooth curve to connect the gap(s) that existed around the lesion boundary. The final contours made sure that lesion locations in strain images should have good correspondence to these in B-mode images. However, achieving similar lesion morphology between the B-mode and strain images was attempted by the operator. It is worth noting that improved delineation of breast masses could be obtained with a board-certified radiologist.

## 3. Results

### 3.1. FEA Results

**Figure 2:**
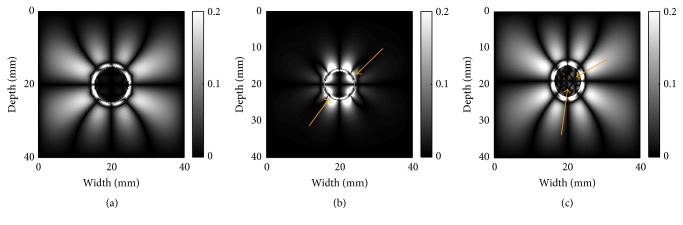
Images of calculated NSDI: (a) a 10 mm inclusion bonded to the background (Case 1), (b) a 10 mm inclusion loosely connected to the background (Case 1), and (c) an 8 mm inclusion bonded to the background (Case 2). Arrows in (b) point to high NSDI values around the slipping interface between the inclusion and the background, while the arrow in (c) points to the high NSDI values inside the inclusion.

Figures 2(a) and 2(b) present images of the NDSI obtained around a fully connected and a loosely connected (friction coefficient of 0.1) inclusion. Regardless of the simulated connectivity, the high concentration of NSDI was observed around the interface between the inclusion and the background. Comparing Figure 2(a) with Figure 2(b), we found that the estimated NSDI was higher around the interface in the case of the loosely bonded inclusion and the high NDSI values spread both inward and outward from the interface. In the case of the fully bonded inclusion, the high NSDI values only spread outward from the interface. The overall pattern of the NDSI distribution in Figures 2(a) and 2(b) was symmetric given the circular inclusion. When the tissue heterogeneity (Figure 2(c)) was included, “packets” of high NSDI values occurred within the inclusion (Figure 2(a) versus Figure 2(c)) on the NSDI image, thereby suggesting that the NDSI could be a tool for visualization of breast lesion heterogeneity.

**Figure 3:**
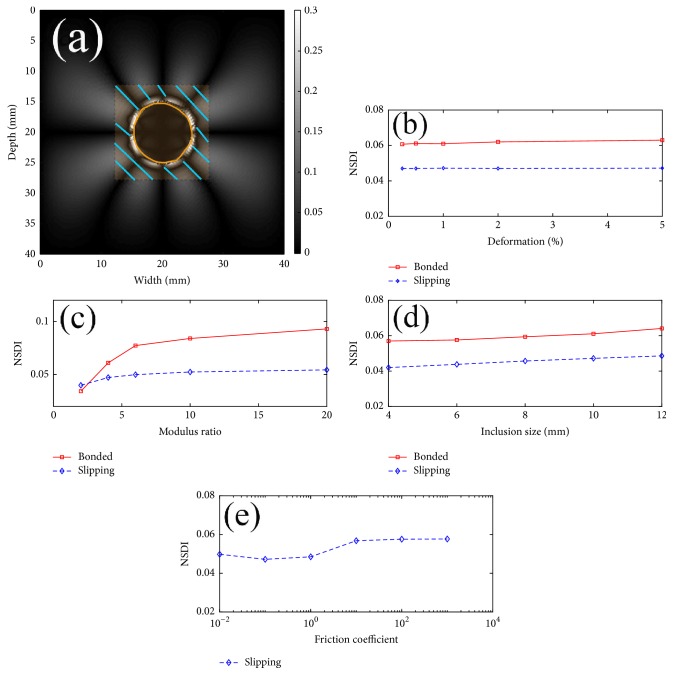
(a) An image illustrating how NSDI was calculated around a 10 mm inclusion (Case 1: modulus ratio of 4, bonded interface, and 1% deformation). The inclusion was delineated by the manually segmented contour in orange color, while the rectangular shaded region centered around the inclusion was calculated by a computer program. The rectangular shaded area outside the inclusion had the same area as that of the inclusion. Four NSDI plots are calculated for (b) Case 1, (c) Case 3, (d) Case 4, and (e) Case 5.

Mean values of the NDSI were calculated within the shaded region of interest (ROI; see Figure 3(a)) around the inclusion for 4 cases investigated (Cases 1 and 3–5). Of note, the shaded ROI had the same size as the size of the inclusion. Figure 3(b) shows that the mean NSDI values at different values of applied compression remained stable. However, the calculated mean NSDI values considerably increased with the increase of the modulus ratio as shown in Figure 3(c). This increasing trend was more obvious in the fully bonded condition. The estimated mean NSDI values were plotted out when the inclusion size increased from 4 mm to 12 mm in Figure 3(d). The calculated NSDI only slightly changed with different levels of compression (3% or less) and with the increase of inclusion size (approximately 12–15%). Also, this trend was not dependent on the connectivity between the inclusion and the background. We also found that the change of the friction coefficient (as an indicator of the connectivity between the inclusion and the background) had little (10% or less) influence over the mean NSDI values (Figure 3(e)). In Figure 3(e), the small fluctuation that occurred when the friction coefficient was around 1 was largely due to the fact that the finite element solution of contact mechanics is a high nonlinear process [28].

**Figure 4:**
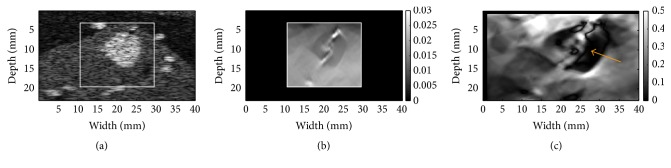
Results from a complex numerical breast phantom (i.e., lesion 2 phantom in a previous publication [22]): (a) a B-mode image simulated by Field II where a rectangular box depicts a ROI, (b) an FE-simulated axial strain image, and (c) an NSDI image within the ROI. In (c), arrow points to the suspected artifact due to the presence of the duct structure.

In the 3D complex breast phantom (see Figure 1(b)), the boundary of the simulated tumor was clearly visible in both the axial strain image (Figure 4(b)) and the NSDI image (Figure 4(c)). We also found that areas with high NSDI values located close to these tissue interfaces (see the tumor-glandular tissue boundary and the glandular-fat interface in Figure 1(b)). In Figure 4(c), the simulated ductal structure was visible in the NSDI images.

### 3.2. *In Vivo* Results

**Figure 5:**
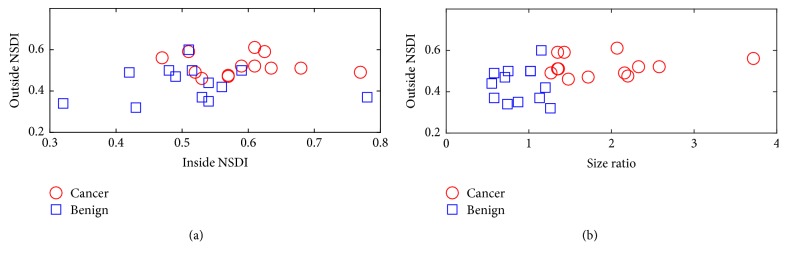
Scatter plots: (a) inside NSDI versus outside NSDI and (b) size ratio versus outside NSDI from 26* in vivo* breast lesions. The size ratio is the lesion size measured from the axial strain elastogram over the lesion size measured from the corresponding B-mode image.

NSDI values were calculated within the corresponding segmented lesions and outside the respective lesions (i.e., an area outside the lesion whose size was equal to the corresponding lesion size; see Figure 3(a)). Of note, the lesion segmentation was conducted on respective axial strain elastograms. Hereafter, we differentiate NDSI values calculated from inside and outside the lesion. They are referred to as the inside NDSI value and outside NDSI value, respectively. A scatter plot displaying the outside NSDI against the inside NSDI is shown in Figure 5(a). As consistent with the scatter plot, based on the Wilcoxon rank-sum test, both the outside and inside NSDI values were significantly lower among benign breast tumors as compared to these among malignant breast cancers (*p* < 0.001 and *p* = 0.025, resp.). Furthermore, the other scatter plot showing the outside NSDI with respect to the size ratio (defined as the lesion size measured from the axial strain elastogram over the lesion size obtained from the corresponding B-mode image) is shown in Figure 5(b). Visually, combining the outside NSDI and the size ratio [4, 5] can separate breast lesions into two clusters, showing good promise.

**Figure 6:**
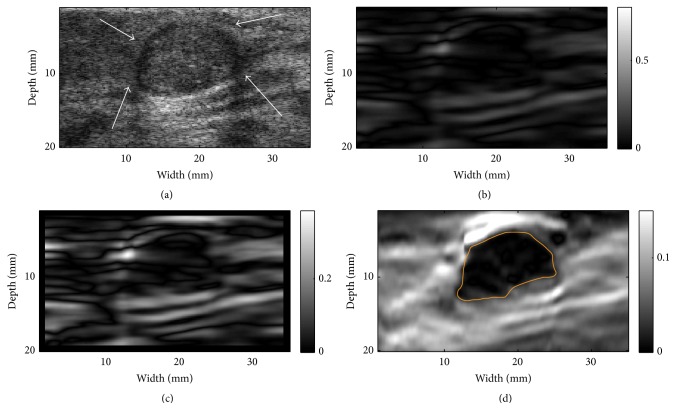
Resultant images of a fibroadenoma (FA): (a) A B-mode image indicating the lesion (see arrows), (b) an NSDI image, (c) a shear strain elastogram, and (d) an axial strain elastogram. The contour on (d) is the segmented target boundary and was used for calculations of NSDI for this case.

**Figure 7:**
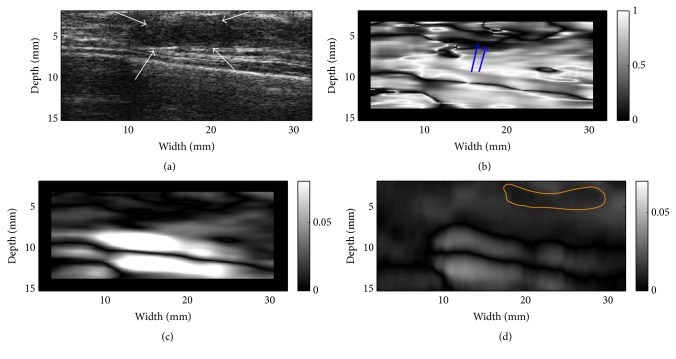
Resultant images of an invasive ductal carcinoma (IDC): (a) A B-mode image indicating the lesion (see arrows), (b) an NSDI image, (c) a shear strain elastogram, and (d) an axial strain elastogram. The contour on (d) is the segmented target boundary and was used for calculations of NSDI for this case. Double arrows in (b) point to the suspected ductal-like structure.

**Figure 8:**
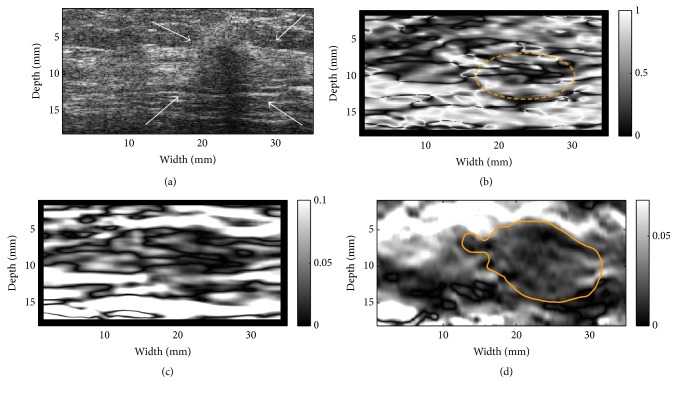
Resultant images of a (unspecified) breast cancer: (a) A B-mode image indicating the lesion (see arrows), (b) an NSDI image, (c) a shear strain elastogram, and (d) an axial strain elastogram. The contour on (d) is the segmented target boundary and was used for calculations of NSDI for this case. The elliptic contour in (b) depicts complex NSDI patterns likely induced due to the heterogeneity.

Three representative examples (one fibroadenoma [FA], one invasive ductal carcinoma [IDC], and one unspecified cancer) were provided in Figures 6–8, respectively. Notably, the outside NSDI values around the FA were considerably lower than these seen around the IDC (Figure 6(b) versus Figures 7(b) and 8(b)). It is also interesting to note that, in 3 out 9 IDC cases, the duct-like structure was visible in the NSDI image (see Figure 7(b)). In the IDC case, the lesion boundary in the shear strain image (Figure 7(c)) was better visualized, whereas the lesion boundary in the axial strain elastogram (Figure 7(d)) was barely visible. In the case of the unspecified breast cancer (i.e., Figure 8), the oscillation of high and low values of NSDI can be seen in Figure 8(b). We stipulate that this is likely due to the tissue heterogeneity as demonstrated by the simplified finite element model (see Figure 2(c)).

## 4. Discussions

Typically, host stromal responses to the aggressive invasion of carcinomas stimulate the pervasive growth of dense fibrous tissue around the tumor (also known as desmoplastic reaction [29]), probably causing a spatial distribution of heterogeneous and significantly hardened stroma. A recent elastography study conducted by Liu et al. [24] demonstrated that malignant masses have more heterogeneous distributions of tissue modulus, as compared to benign ones. Also, the invasion of cancerous cells tends to follow “specific” low resistance directions around the cancer-stromal interface, and this pattern of growth leads to “stellate” appearance [30], probably causing malignant cancers to firmly connect to their surrounding tissues [31]. This firm connection could cause malignant tumors to be less mobile as compared to benign ones. Consequently, these biological implications could be used to justify the existence of firm connectivity and stiffness heterogeneity among malignant breast cancers. As we learned from the FEA experiment (see the summary in Table 2), these two factors led to high outside and inside NSDI values.

Many clinical studies in breast SE [6, 7] have been often performed using axial strain elastogram data. Our result suggested that additional information such as shear strain elastogram and the NSDI image may provide useful information. For instance, both our FEA simulation and* in vivo* experiment indicated that the NSDI could depict the duct-like structure, which could be an indication of IDC. Furthermore, the shear strain elastogram (see Figure 7(c)) may depict the tumor boundary better as compared to the axial strain elastogram. The axial strain elastogram showed the low contrast between the IDC lesion and its background and this could be attributed to the nonlinear tissue elasticity [5, 26], though the exact reason is not known.

Several factors could potentially confound local shear deformation. In addition to the nonlinear elasticity [32], the slipping boundary in the tumor-background interface could be another confounding factor because it could cause high NSDI values around the tumor boundary (see Figure 2(b)). Tissue-dependent viscosity could also play a role in the change of strain contrast, thereby affecting the shear deformation. In this preliminary study, our FEA simulations were mainly limited to linearly elastic materials and we consider this as a limitation. We noticed that the complex breast model provided more realistic NSDI images as compared to those simplistic models (i.e., Cases 1–5). Hence, the available open-source elastography simulator [22] will be used to study above-identified confounding factors in future numerical studies. Advanced imaging simulations are ideally suited because they are readily available and the cost is low.

Another limitation is the small number of cases investigated. Given the fact that only 26* in vivo* breast tumors were studied, more sophisticated statistical analyses were left for future studies. Outcomes of our future studies could be further improved because we are planning on using an optimal frame selection technique [33, 34] to optimize data selection. It is also worth noting that locations of all 26 biopsy-confirmed breast lesions were identified by an experienced biomedical engineer. Although these lesion boundaries were largely consistent with these delineated by board-certified radiologists in an early study [7], the exact tumor boundaries registered with respective pathology were not available for this study.

As shown in Figure 5(a), we also want to note that both the inside and outside NSDI values were elevated in the majority of breast cancers. This observation could be useful for breast lesion differentiation. However, this study was not designed to demonstrate the clinical utility of NSDI for two reasons. First, the quality of lateral displacement estimates after the denoising was relatively poor as compared to these axial displacement estimates. Because of that, the estimation uncertainty of NSDI is still relatively high. In the future, the utility of novel beamforming-based techniques [35–37] may significantly improve the quality of lateral displacement estimation. Thus, we are optimistic that the combination of our denoising approach with one of these beamforming methods should significantly improve the estimation of local shear deformation. Second, in order to accurately estimate local shear deformation, displacements in all three dimensions are needed. Certainly, the definition of NSDI should be modified accordingly. With the availability of whole breast ultrasound scanning systems, obtaining* in vivo* 3D ultrasonically measured displacement estimates becomes feasible [38]. Therefore, a large clinical study of the NSDI is still in the planning stage. Nevertheless, we still feel it appropriate to make one intriguing, albeit subjective, observation regarding the feasibility of the proposed NSDI metric.

In this work, we described the options we have chosen and gave justifications for those choices. While we believe that they are good choices, combining a high-quality subsample estimation method with denoising represents, however, only a feasible path to calculate the proposed NSDI metric. Other paths are also possible. For instance, the above-mentioned novel beamforming methods [35–37] alone may be able to provide high-quality lateral displacement estimates that can be used to calculate the NSDI metric.

## 5. Conclusions

The proposed NSDI metric was evaluated using FEA models and* in vivo* ultrasound data. This feasibility study showed that the elevated NSDI values should theoretically be correlated to two factors accompanying malignant breast cancers: firm connectivity and stiffness heterogeneity. Initial results also suggest that statistically significant differences in the inside and outside NSDI values were found between the benign and malignant breast tumors. In summary, our preliminary results demonstrated that this conceptually and computationally simple method could be used to improve ultrasound SE with current clinical equipment. Further studies, particularly in conjunction with the 3D ultrasound data, are being planned to explore the clinical utility of the proposed method.

## Figures and Tables

**Table 1:** Descriptions of 5 simulated cases. The modulus ratio is the shear modulus ratio between the inclusion and background.

Case number	Modulus ratio	Inclusion size	Background-inclusion interface	Deformation level	Other information
1	4	10 mm	Bonded and slipping (friction coefficient = 0.1)	0.25%–5%	Plane strain
2	5	10 mm	Bonded	1%	Smaller targets within the inclusion
3	2–20	10 mm	Bonded and slipping (friction coefficient = 0.1)	1%	Plane strain
4	4	4–12 mm	Bonded and slipping (friction coefficient = 0.1)	1%	Plane strain
5	4	10 mm	Varying slipping condition (friction coefficient = [0.1 1000])	1%	Plane strain

**Table 2:** A summary of observations through the FEA study.

Condition	Description	Observation
1	High modulus ratio between the inclusion and the background	High mean outside NSDI

2	Heterogeneity within the inclusion	High inside NSDI

3	Slipping boundary between the inclusion and the background	High NSDI values around the inclusion-background interface
